# PD-1 Inhibitor Combined With Radiotherapy and GM-CSF (PRaG) in Patients With Metastatic Solid Tumors: An Open-Label Phase II Study

**DOI:** 10.3389/fimmu.2022.952066

**Published:** 2022-07-08

**Authors:** Yuehong Kong, Xiangrong Zhao, Meiling Xu, Jie Pan, Yifu Ma, Li Zou, Qiliang Peng, Junjun Zhang, Cunjin Su, Zhi Xu, Wei Zhou, Yong Peng, Jiabao Yang, Chengliang Zhou, Yujia Li, Qiuchen Guo, Guangqiang Chen, Hongya Wu, Pengfei Xing, Liyuan Zhang

**Affiliations:** ^1^ Department of Radiotherapy & Oncology, The Second Affiliated Hospital of Soochow University, Suzhou, China; ^2^ Institution of Radiotherapy & Oncology, Soochow University, Suzhou, China; ^3^ Laboratory for Combined Radiotherapy and Immunotherapy of Cancer, The Second Affiliated Hospital of Soochow University, Suzhou, China; ^4^ Department of Pharmacy, The Second Affiliated Hospital of Soochow University, Suzhou, China; ^5^ Medical Affairs, ICON Public limited company (ICON Plc), Beijing, China; ^6^ Department of Radiology, The Second Affiliated Hospital of Soochow University, Suzhou, China; ^7^ Jiangsu Institute of Clinical Immunology, The First Affiliated Hospital of Soochow University, Suzhou, China; ^8^ Suzhou Key Laboratory for Tumor Immunology of Digestive Tract, Suzhou, China

**Keywords:** radiotherapy, tumor microenvironment, PD-1 inhibitor, granulocyte macrophage-colony stimulating factor, chemotherapy refractory

## Abstract

Patients with metastatic cancer refractory to standard systemic therapies have a poor prognosis and few therapeutic options. Radiotherapy can shape the tumor microenvironment (TME) by inducing immunogenic cell death and promoting tumor recognition by natural killer cells and T lymphocytes. Granulocyte macrophage-colony stimulating factor (GM-CSF) was known to promote dendric cell maturation and function, and might also induce the macrophage polarization with anti-tumor capabilities. A phase II trial (ChiCTR1900026175) was conducted to assess the clinical efficacy and safety of radiotherapy, PD-1 inhibitor and GM-CSF (PRaG regimen). This trial was registered at http://www.chictr.org.cn/index.aspx. A PRaG cycle consisted of 3 fractions of 5 or 8 Gy delivered for one metastatic lesion from day 1, followed by 200 μg subcutaneous injection of GM-CSF once daily for 2 weeks, and intravenous infusion of PD-1 inhibitor once within one week after completion of radiotherapy. The PRaG regimen was repeated every 21 days for at least two cycles. Once the PRaG therapy was completed, the patient continued PD-1 inhibitor monotherapy until confirmed disease progression or unacceptable toxicity. The primary endpoint was objective response rate (ORR). A total of 54 patients were enrolled with a median follow-up time of 16.4 months. The ORR was 16.7%, and the disease control rate was 46.3% in intent-to-treat patients. Median progression-free survival was 4.0 months (95% confidence interval [CI], 3.3 to 4.8), and median overall survival was 10.5 months (95% CI, 8.7 to 12.2). Grade 3 treatment-related adverse events occurred in five patients (10.0%) and grade 4 in one patient (2.0%). Therefore, the PRaG regimen was well tolerated with acceptable toxicity and may represent a promising salvage treatment for patients with chemotherapy-refractory solid tumors. It is likely that PRaG acts *via* heating upthe TME with radiotherapy and GM-CSF, which was further boosted by PD-1 inhibitors.

## Introduction

Immunotherapy, in particular programmed cell death protein-1/programmed cell death ligand-1 (PD-1/PD-L1) blockade, significantly changed treatment paradigms in oncology and achieved considerable therapeutic efficacy across major types of solid tumors (1). However, the majority of patients did not respond to single-agent PD-1/PD-L1 blockade therapy, and the objective response rate (ORR) was only about 15–25% in most solid tumors such as non-small cell lung cancer (NSCLC), head and neck, gastroesophageal, bladder, and urothelial cancers (1). Additionally, in the second line or above treatment for patients with various metastatic cancers, particularly for patients with PD-L1 negative or microsatellite stability (MSS)/mismatch repair deficiency (dMMR) or low tumor mutation burden (TMB), the efficacy of single-agent PD-1/PD-L1 inhibitors were even much lower (2). It is thus challenging but urgently needed to provide beneficial treatment options for patients insensitive to single-agent immunotherapy. Several recent studies aimed to investigate the potential synergistic effects of PD-1/PD-L1 blockade therapy with radiotherapy in patients with advanced cancer (3–5), however it remains uncharacterized whether PD-1/PD-L1 blockade therapy, radiotherapy could also be combined with other immunomodulating strategy to achieve maximal therapeutic efficacy particularly for those cancer patients with advanced and metastatic diseases.

Radiotherapy has been shown to stimulate the antitumor immune response and might synergize with PD-1/PD-L1 inhibitors (4, 6–8). Technological advances enable the delivery of higher doses of localized radiation to tumor targets, which would be a potentially curable approach for oligometastatic disease and effective treatment for multiple metastatic cancer (9, 10). High-dose radiotherapy (≥5 Gy) often results in the tumor microenvironment (TME) modulation such as inducing an immunostimulatory form of cell death, called immunogenic cell death (ICD) (3, 11). In this regard, irradiation can uncover or release previously hidden antigens and trigger remarkable immune-stimulatory effects, such as enhancing the expression of MHC-I on the tumor cell surface, upregulating FAS/CD95, normalizing aberrant tumor vasculature, and promoting the release of cytokines and chemokines, which can improve the infiltration of multiple immune cells into the tumor (8, 12–14). These synergistic effects ultimately lead to the recruitment and priming of immune effector cells in the TME, resulting in significant antitumor activities in local irradiated, and distant unirradiated tumors (12–14).

Radiotherapy can upregulate PD-L1 expression (4, 15), and emerging evidence has shown clinical efficacy of radiotherapy in combination with pembrolizumab in metastatic NSCLC (16–18), especially in those PD-L1 negative subgroups (17). Furthermore, chemo-radiotherapy (CRT) can increase TMB and dysregulation of MMR system-related genes, thus altering MSI status (19). For instance, in a phase II trial, MSS colorectal and pancreatic adenocarcinoma patients, considered insensitive to anti-PD-1 immunotherapy, could benefit from the combined radiotherapy and immunotherapy (20). Notably, irradiation to most metastatic sites might be more effective when combing with immunotherapy (10, 21).

In addition, granulocyte-macrophage colony-stimulating factor (GM-CSF) may display multiple immunostimulatory activities such as improved dendritic cells (DCs) function and further augment the anti-tumor effects with radiotherapy or PD-1 inhibitors (22, 23). Given that PD-1 inhibitors, radiotherapy, and GM-CSF (PRaG regimen) act specifically *via* distinct components of the cancer-immunity cycle, we aim to assess the clinical efficacy of triple-combination therapy in chemo-refractory patients with metastatic solid tumors. To this end, we conducted a phase II study in which the three modalities, PD-1 inhibitor, GM-CSF, and radiotherapy, were sequentially administered. And we believe that repeated cycles of radiotherapy in combination with GM-CSF might have multiple immune-stimulatory effects and reduce tumor burden, which possibly maximize a continuously synergistic effect with PD-1 inhibitors. To our knowledge, our present study provided the first evidence of efficacy and safety of multi-cycles of PRaG regimen in patients with chemotherapy-refractory solid tumors.

## Patients and Methods

### Patients

Patients ≥18 years old with chemo-refractory metastatic solid tumors and exhausted standard treatment and Eastern Cooperative Oncology Group performance status (ECOG)≤3 were eligible for enrollment. A patient must have at least three measurable lesions by Response Evaluation Criteria in Solid Tumors version 1.1 (RECIST v1.1) and at least two lesions amenable for radiotherapy. Patients were required to have adequate organ function, including absolute neutrophil count ≥1,500/mL, serum creatinine level ≤1.5 upper limit of normal (ULN), AST and ALT ≤2.5 ULN (or ≤5 ULN for patients with liver metastases), and albumin level ≥3.5mg/dL. Details of inclusion and exclusion criteria are available in **Supplement 1**.

### Study Design and Interventions

This was an investigator-initiated, single-center (The Second Affiliated Hospital of Soochow University), single-arm, open-label, phase II study to evaluate the clinical efficacy and safety of PRaG regimen in patients with chemo-refractory metastatic solid tumors. The study protocol and amendments were approved by the Ethics Committee of The Second Affiliated Hospital of Soochow University. All patients signed written informed consent before enrollment. This trial was registered at http://www.chictr.org.cn/index.aspx (ChiCTR 1900026175).

**Figure 1:**
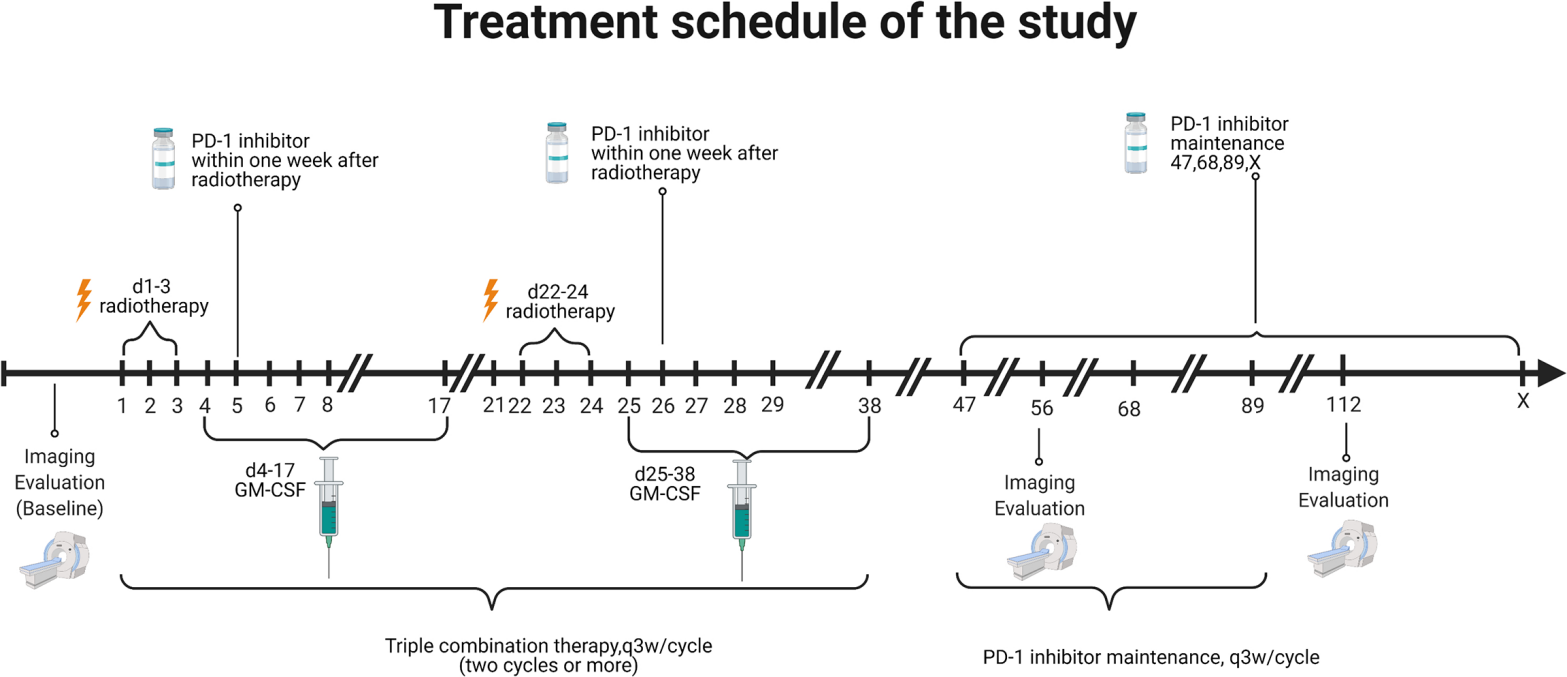
Treatment schedule of the study.

Eligible patients received at least two cycles of PRaG. In a PRaG cycle, stereotactic body radiotherapy (SBRT) or hypofractionated radiotherapy (HFRT; 5 Gy or 8 Gy × 3 fractions) was delivered for one unirradiated metastatic lesion on day 1, followed by GM-CSF 200 μg subcutaneous injection daily for 2 weeks starting within 24 h after the completion of radiotherapy. An anti-PD-1 antibody was intravenously administered within 1 week after completion of radiotherapy. PRaG was repeated every 21 days for at least two cycles until there were no appropriate lesions for irradiation or reaching the tolerance dose of normal tissues. Patients who completed or discontinued PRaG for reasons other than immune-related adverse events and without confirmed disease progression proceed with a PD-1 inhibitor (**Supplement 1**) as maintenance monotherapy until clinical or radiographic disease progression or unacceptable toxicity. Treatment was allowed through disease progression until confirmed disease progression, unacceptable toxicity, or loss of clinical benefit as judged by the investigator. The patient was not allowed to receive other systemic anticancer therapies during treatment. The treatment scheme is illustrated in **Figure 1**. The details of the treatment regimen are available in **Supplement 1**.

Symptomatic or clinically relevant metastasis was prioritized when selecting the irradiation sequence of metastases. Radiotherapy was delivered using photons with linear accelerators once daily at 8 Gy for three fractions for each lesion. If the irradiated target was close to hollow organs or other safety considerations, the fractionation was allowed to adjust to 5 Gy for three fractions.

### Efficacy and Safety Assessment

Adverse events (AEs) were collected from the time patient signed informed consent forms (ICF) until 90 days after the last administration of study treatment. AEs were graded according to Common Terminology Criteria for Adverse Events (CTCAE), version 4.0, and the investigator assessed its causality. The highest severity and level of the cause of AEs were reported. Tumor response was evaluated by an independent radiologist review of enhanced computed tomography scan or magnetic resonance imaging scan scheduled every eight weeks according to RECIST v1.1. The target lesions were selected before treatment for evaluation and were not allowed for radiotherapy.

### Endpoints

The primary endpoint was objective response rate (ORR) in intention­to­treat (ITT) patients by RECISIT v1.1. Secondary endpoints included safety, disease control rate (DCR), progression-free survival (PFS), and overall survival (OS). ORR was defined as the proportion of patients with complete response (CR) or partial response (PR). DCR was defined as the percentage of patients with CR, PR, or stable disease (SD) from enrollment. OS was calculated from the enrollment date to the date of death or last known alive. PFS was calculated from the enrollment date to disease progression, death, or censored at the last clinical follow-up. The nature, frequency, and severity of adverse events were assessed based on CTCAE 4.0. Lymphocyte subset counts and cytokine analysis were examined as exploratory endpoints.

### Flow Cytometry

Blood samples were collected before and after each treatment cycle. Peripheral venous blood (2 mL) was placed in an EDTA anticoagulant tube for mixing. The detection was carried out according to the direct immunolabeling method. The erythrocytes were lysed by the Optilyse procedure, and then 20 μL of mixed color fluorescent antibody reagent of anti-CD3, anti-CD4, anti-CD8, anti-CD19, anti-CD16, and anti-CD56 (BD Biosciences, USA) samples were added into 50 μL of fully mixed blood samples. The samples were incubated in the dark at room temperature for 15 - 20 minutes, and 450 ul of hemolysin was added to lyse red blood cells in the dark at room temperature for 10 minutes. Then data were acquired by FACS Canto (Becton Dickinson, CA) and the acquired data were analyzed with Flowjo 8 software.

### Cytometric Beads Array

Blood samples before and after each treatment cycle were collected. Collected 5 mL of venous blood, placed it in an ordinary vacuum tube without anticoagulant, warmed it in a water bath for 30 min, centrifuged at 3,500 rpm for 10 min, and took the upper serum. Prepared 2 mL diluent to reconstitute the standard and diluted with 1:2, 1:4, 1:8, 1:16, 1:32, 1:64, 1:128, and 1:256 times ratio. The reagents were IL-2, IL-4, IL-6, IL-10, IL-17A, TNF, and IFN-γ capture microsphere tubes (Becton Dickinson, CA), and captured microspheres according to the detection technique including standards and quality control calculation, fully shaken and mixed each tube of microspheres, and sucked them out into one tube, labeled “Mixed capture microspheres”. Centrifuged the mixed capture microspheres at 200×*g* for 5 min, discarded the supernatant, and transferred 50 uL of the standards of different concentrations to the corresponding quality control tubes. Added 50 μL of mixed capture microspheres to each detection tube, added 50 μL of the sample, added PE-labeled detection antibody, and incubated for 2 h at room temperature in the dark. Added 1 mL washing solution, centrifuged at 200×*g* for 5 min, discarded the supernatant, added 300 μL washing suspension weight PCM to be tested, and used FACS instrument for testing (Becton Dickinson, CA).

### Statistical Analysis

This phase II study was not designed to test a specific hypothesis around the primary end point. A sample size of 50 patients was chosen to provide relatively certain level of precision as described in **Supplement 1**.

Normally distributed variables with equal variance were compared with one-way ANOVA among CR+PR, SD, and PD groups. The Kruskal–Wallis test was used to compare numerical variables either with abnormal distribution or unequal variance among the three groups. Categorical variables were described with *n* (%) and compared with the Chi-square test or Fisher’s exact test among the CR+PR, SD, and PD groups. A general linear model (GLM) was used to analyze prognostic factors at different time points among the three groups. The PFS and OS endpoints in ITT patients were estimated with the Kaplan–Meier method. The *p* value less than 0.05 (two-sided) was considered statistically significant. PASS 15 was used for sample size calculation and SPSS 22 for statistical analyses.

## Results

### Patient Characteristics

**Figure 2:**
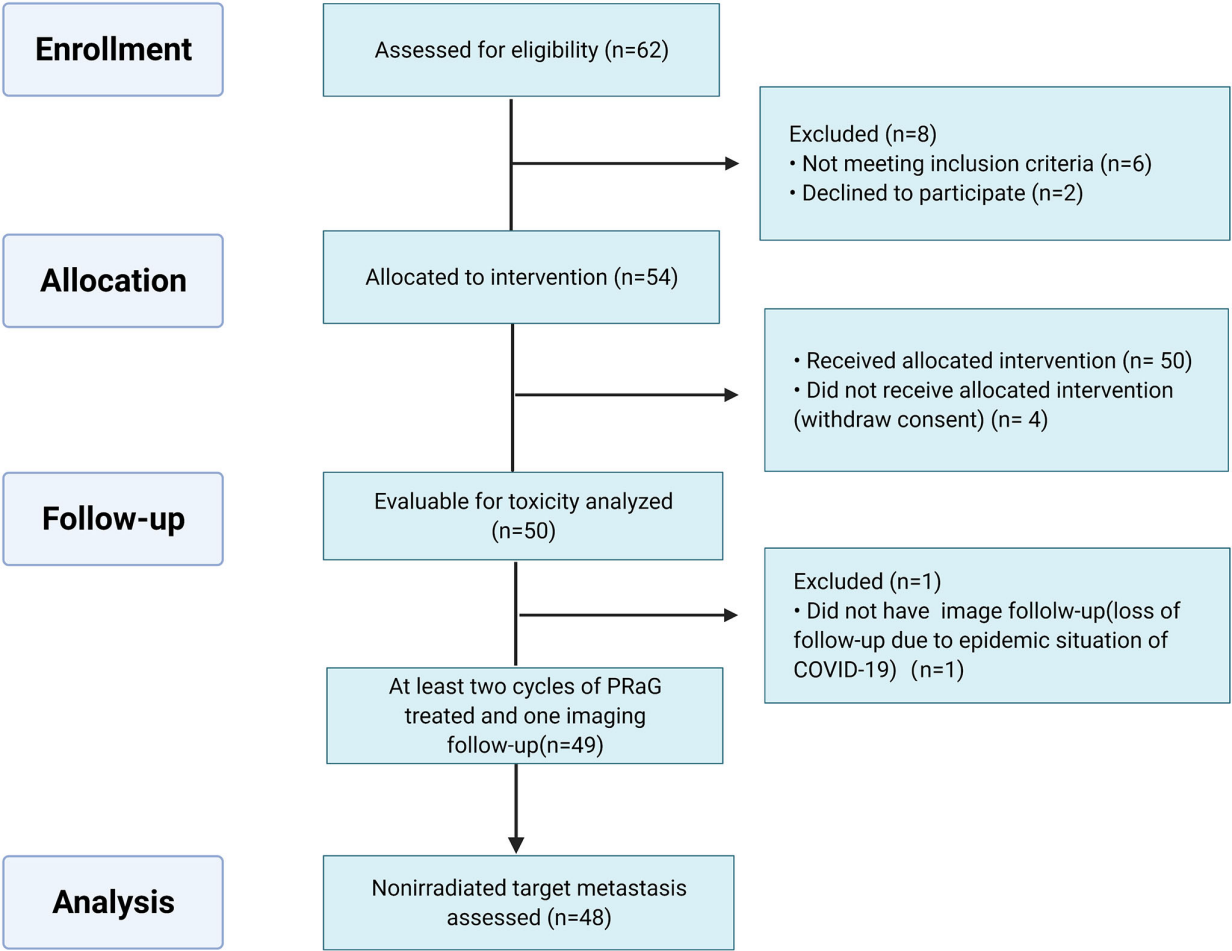
Consort diagram.

Between March 2019 and December 2021, 54 patients were enrolled in the ITT population. The median age of the patients was 60 years old (range: 31–76 years). Most of the patients enrolled in this study were heavily treated with a median of three prior lines of systemic therapies (range: 1–9). Thirty-nine patients (72.2%) had poor ECOG performance status of 2, 3. Five patients (9.3%) failed previous anti-PD-1/PD-L1 therapy before enrollment. Thirty patients (55.6%) had more than five metastatic lesions, and eighteen patients (33.3%) had more than ten metastases. The most common sites of metastasis were lymph nodes (44.4%), liver (31.5%), lung (29.6%), bone (27.8%), pleuroperitoneum (13.0%), and brain (11.1%). Of the patients, 62.7% had two or fewer organs of metastasis, and 37.3% of patients had more than two sites (median, 2 sites; range, 1 to 5 sites). The baseline characteristics of the 54 patients are summarized in **Table 1**. Fifty patients were evaluable for safety and forty-eight patients for efficacy (**Figure 2**).

**Table 1:** Patient characteristics.

Characteristic	No.
Age, Median, range (years)	60 (31–77)
Gender	
Male	25 (46.3%)
Female	29 (53.7%)
ECOG performance status	
0	1 (1.9%)
1	14 (25.9%)
2	32 (59.3%)
3	7 (13.0%)
No. of prior systemic therapies	
1	2 (3.7%)
2	17 (31.5%)
3	19 (35.2%)
≥4	16 (29.6%)
Prior PD-1/PD-L1 inhibitor	
Yes	5 (9.3%)
No	49 (90.7%)
No. of metastatic site	
≤5	24 (44.4%)
5-10	12 (22.2%)
≥10	18 (33.3%)
Metastatic organs involved	
1	15 (27.7%)
2	19 (35.2%)
3	13 (24.1%)
≥4	7 (13.0%)
PD-L1	
<1%	15 (27.8%)
≥1%	17 (31.5%)
MMR	
MSS	14 (25.9%)
MSI-L	1 (1.8%)
Primary cancer sites	
Lung	13 (24.1%)
Colorectum	8 (14.8%)
Breast	5 (9.3%)
Gastro	5 (9.3%)
Cervix	4 (7.4%)
Esophagus	4 (7.4%)
Ovary	4 (7.4%)
Head and neck	4 (7.4%)
Liver	2 (3.7%)
Others*	5 (9.3%)
*Bile duct 1 (1.8%), vulva 1 (1.8%), kidney 1 (1.8%), soft tissue 2(3.7%)	
metastatic tumor sites	
Lymph nodes	24 (44.4%)
Bone	15 (27.8%)
Lung	16 (29.6%)
Liver	17 (31.5%)
Brain	6 (11.1%)
Pleuroperitoneum	7 (13.0%)
Other sites	9 (16.7%)
Pancreas 2 (3.7%), skin 1 (1.8%), breast 1 (1.8%), abdominal wall 1(1.8%), muscle 1(1.8%), Thyroid 1(1.8%), Appendix 1(1.8%),spleen 1(1.8%)	
irradiated tumor sites	
Lymph node	23 (42.6%)
Lung	15 (27.8%)
Liver	13 (24.1%)
Bone	11 (20.4%)
Brain	8 (14.8%)
Chest wall	4 (7.4%)
Other sites*	7 (13.0%)
*Breast 1 (1.8%), diaphragm 1 (1.8%), abdominal wall1(1.8%),Stomach 2(3.8%),Rectum 1(1.8%), Vaginal stump 1(1.8%)	

### Treatment

As of the data cutoff date of December 31st, 2021, the median duration of follow-up was 16.4 months (range: 1.7–33.1 m). Four patients have withdrawn consent and one patient received only one cycle of PRaG and discontinued from the study due to the epidemic situation of COVID-19. Forty-nine patients received at least two cycles of PRaG with a median of four PRaG cycles (range: 2–12) and had at least one imaging follow-up. Forty-eight patients were evaluable for nonirradiated target lesions. The median number of total treatment cycles (PRaG cycle and PD-1 inhibitor maintenance cycle) was six (range: 2–16). In a total of 214 PRaG cycles, RT of 8 Gy × 3 fractions were delivered in 145 (67.8%) cycles. A low dose of 5 Gy × 3 fractions was delivered in 68 cycles (31.8%). Except for one patient who had RT dose modification to 8 Gy × 2 fractions due to irradiation target in the previously radiated field, there was no other adverse event leading to RT dose and fractionation reduction. The median number of irradiated sites was three (range: 1–9),and the most common irradiated tumor sites were lymph nodes in 23 patients (42.6%), followed by lung lesions in 15 patients (27.8%), liver lesions in 13 patients (24.1%), bone lesions in 11 patients (20.4%), brain lesions in 8 patients (14.8%), and chest wall metastases in 4 patients (7.4%).

### Efficacy

**Figure 3:**
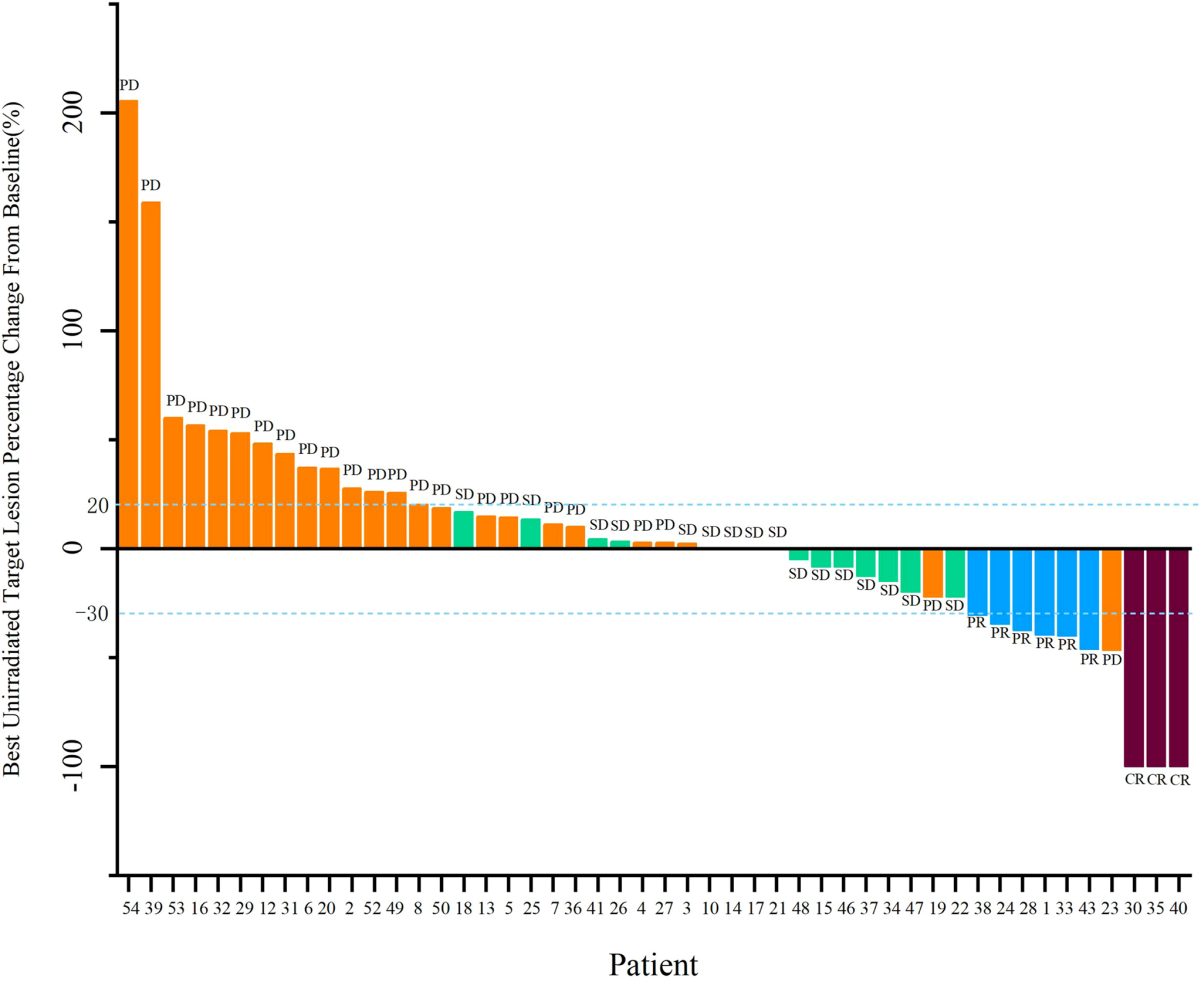
Waterfall plots of maximum percent change in nonirradiated RECIST target lesions.

**Figure 4:**
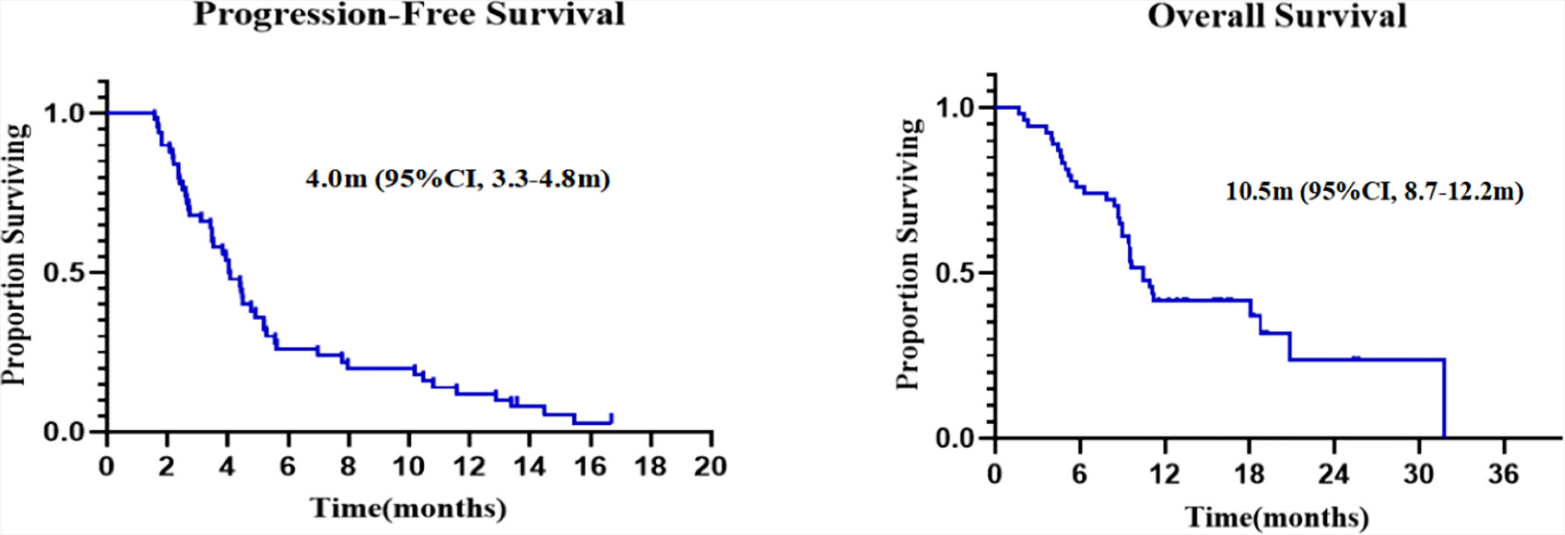
Kaplan–Meier curves of progression-free survival and overall survival.

Forty-eight patients with at least one nonirradiated site assessment after treatment were included in the response-evaluable population. In response-evaluable patients, three patients had confirmed complete response (CR), six patients had confirmed partial responses (PR), 16 patients had stable diseases (SD), and 23 patients experienced progressive disease (PD; **Figure 3**). The ORR was 18.8% and DCR was 52.1% in evaluable patients. The ORR was 16.7%, and DCR was 46.3% in ITT patients. The three patients who achieved CR included one MSS colon cancer patient, one MSS gastric cancer patient and one driver gene negative (wide type for EGFR, ALK, ROS1, and BRAF) NSCLC patient with PD-L1 combined positive score (CPS) of 30. The NSCLC patient who had progressed on previous first line chemotherapy and second line combinational pembrolizumab with bevacizumab achieved CR since cycle 8 and maintained for 15 months by data cut-off. Tumor response by primary tumor was shown in **Supplementary Table 2**. Median PFS was 4.0 months (95% CI, 3.3 to 4.8), and median OS was 10.5 months (95% CI, 8.7 to 12.2; **Figure 4**).

### Safety

All the treatment-related adverse events (TRAEs) reported in the safety analysis are shown in **Table 2**. TRAEs of any grade occurred in 35 (70.0%) patients, the most common TRAEs were fatigue (66.0%), anorexia (48.0%), fever (38.0%), and thyroid dysfunction (30.0%). There was no grade 5 TRAE. Grade 3 and 4 TRAEs were observed in 6 subjects (12.0%), which included pneumonitis (grade 3), hepatic toxicity (grade 3), fatigue (grade 3), anorexia (grade 3), fever (grade 3), and pneumonia (grade 4) for each patient, and five patients (10.0%) discontinued the treatment due to TRAEs. The two patients who received irradiation at lung metastases developed pneumonia/pneumonitis during PD-1 inhibitor maintenance therapy. In particular, the patient who developed grade 4 pneumonitis received irradiation to three distinct right lung metastases during three cycles of PRaG. Notably, pneumonitis was localized at the irradiated right lung, which was likely related to radiotherapy with a potential contributing factor that the patient received mediastinum radiotherapy 12 months ago. But the patient also developed pneumonia caused by bacterial infection. The patient discontinued anti-PD-1 therapy and had hormone and antibiotic therapy. The patient with grade 3 pneumonitis presented at the irradiated right lung and the non-irradiated left lung, was related to PD-1 inhibitor. The grade 3 hepatic toxicity was related to anti-PD-1 antibody, and the patient suspended immunotherapy, and liver function was recovered after hepatoprotective therapy. Three patients experienced a transient decrease in pulse oxygen saturation after GM-CSF administration and rapidly recovered after oxygen inhalation. Nineteen patients (38.0%) had grade 1–3 fever related to GM-CSF (**Supplementary Table 1**).

**Table 2:** Treatment-related adverse events (TRAEs).

Parameter	Evaluable for toxicity analyzedN=50, No(%)		
Any TRAES		35 (70.0)	
Grade 3		5 (10.0)	
Grade 4		1 (2.0)	
TRAES leading to treatment discontinuation		5 (10.0)	
Deaths		0 (0)	
Patients with TRAEs	Any grade	Grade 3	Grade 4
Fatigue	33 (66.0)	1 (2.0)	0 (0)
Anorexia	24 (48.0)	1 (2.0)	0 (0)
Fever	19 (38.0)	1 (2.0)	0 (0)
Thyroid dysfunction	15 (30.0)	0 (0)	0 (0)
Liver dysfunction	6 (12.0)	1 (2.0)	0 (0)
Rash	7 (14.0)	0 (0)	0 (0)
Vomiting	7 (14.0)	0 (0)	0 (0)
Pneumonia/Pneumonitis	7 (14.0)	1 (2.0)	1 (2.0)
Myocarditis	1 (2.0)	0 (0)	0 (0)
Uveitis	1 (2.0)	0 (0)	0 (0)
Pruritus	2 (4.0)	0 (0)	0 (0)
Decrease in pulse oxygensaturation	3 (6.0)	0 (0)	0 (0)
Leukocytosis	5 (10.0)	0 (0)	0 (0)

### Analysis of Baseline Characteristics and Prognostic Factors

The association of baseline factors and clinical outcomes between the CR+PR, SD, and PD groups were analyzed and shown in **Table 3**. Baseline age and liver metastases were associated with tumor response. Patients with liver metastases (*p* = 0.001) and a lower baseline CD4^+^/CD8^+^ ratio (*p* = 0.026) had worse therapeutic effects when comparing the PD group with the SD group. Furthermore, the irradiated sites, baseline peripheral neutrophil to lymphocyte ratio (NLR), the cycle number of PRaG treatment, and metastatic organs and numbers were not correlated with disease response (*p *> 0.05). Baseline of peripheral lymphocyte subset numbers of three groups (CR+PR, SD, PD) has no significant differences: baseline absolute CD3^+^T cell numbers (*p* = 0.408), CD3^+^CD4^+^T cell (*p* = 0.258), CD3^+^CD8^+^T cell (*p* = 0.343), and CD19^+^B cell (*p* = 0.937; **Table 3**). The changes in lymphocyte subset percentage after treatment from baseline between the three groups (CR+PR, SD, PD) were shown in **Figure 5**. Peripheral CD3^+^T cell, CD3^+^CD4^+^T cell, CD3^+^CD8^+^T cell, and natural killer (NK) cell numbers increased after one cycle of PRaG in the CR+PR group compared with the other two groups (**Supplementary Figure 1**). None of the lymphocyte subset percentage changes showed statistical differences (*p *> 0.05; **Supplementary Figure 2**). The levels of IL-2, IL-4, IL-6, IL-10, TNF, and IFN-γ had no difference among the three groups (**Supplementary Figure 3**).

**Table 3:** Comparison of characteristics between complete response (CR)+partial response (PR), stable disease (SD), and progressive disease (PD) groups after treatment.

Baseline characteristics	CR+PR	SD		PD	*p* value
	(*n* = 9)	(*n* = 16)		(*n* = 23)	
Age, year	63.3 ± 9.9	65.5 ± 8.4		54.0 ± 12.2^*#^	**0.004**
Male, *n* (%)	5(55.6)	7(43.8)		12(52.2)	0.864
ECOG score, *n* (%)					0.304
0	0 (0.0)	0 (0.0)		1 (4.4)	
1	5 (55.6)	2 (12.5)		7 (30.4)
2	3 (33.3)	13 (81.3)		11 (47.8)
3	1 (11.1)	1 (6.2)		4 (17.4)
Number of metastatic lesions	7 (3, 42)	6 (3, 12)		8 (6, 18)	0.412
Number of metastatic organs	2 (1, 2)	3 (2, 3)		2 (2, 3)	0.595
Number of previous systemic therapy	3 (2, 4)	3 (2, 3)		3 (2, 4)	0.49
Lymph nodes metastases, *n* (%)	7 (77.8)	13 (81.3)		15 (65.2)	0.631
Liver metastases, *n* (%)	3 (33.3)	0 (0.0)		13 (56.5)^#^	**0.001** ^a^
PRaG cycles	5 (4, 6)	4 (3, 5)		3(2, 5)	0.2
Irradiation organs, *n* (%)					0.525
Liver	2 (22.2)	0 (0.0)		5 (21.6)	
Lung	3 (33.3)	2 (12.5)		2 (8.7)	
Lymph nodes	3 (33.3)	6 (37.7)		7 (30.4)	
Bone	0 (0.0)	1 (6.2)		3 (13.0)	
Liver+Lung	0 (0.0)	1 (6.2)		1 (4.4)	
Liver+Lymph nodes	0 (0.0)	1 (6.2)		0 (0.0)	
Liver+Bone	0 (0.0)	0 (0.0)		1 (4.4)	
Lung+Lymph nodes	0 (0.0)	0 (0.0)		1 (4.4)	
Lung+Bone	0 (0.0)	1 (6.2)		0 (0.0)	
Lymph nodes+Bone	0 (0.0)	2 (12.5)		1 (4.4)	
Lung+Lymph nodes+Bone	1 (11.2)	0 (0.0)		0 (0.0)	
Others	0 (0.0)	2 (12.5)		2 (8.7)	
Neutrophil to lymphocyte ratio	4.3 ± 4.1	4.4 ± 2.1		4.5 ± 2.4	0.976
Baseline CD3^+^ T cells (cells/ul)	955 ± 546	721 ± 404		751 ± 412	0.408
Baseline CD3^+^CD4^+^T cells(cells/ul)	512 ± 284	424 ± 257		358 ± 200	0.258
Baseline CD3^+^CD8^+^T cells(cells/ul)	420 ± 299	283 ± 188	356 ± 222		0.343
Baseline CD19^+^B cells (cells/ul)	123 ± 67	149 ± 66	113 ± 93		0.937
Baseline CD4^+^/CD8^+^ ratio	1.38 ± 0.62	1.70 ± 0.95	1.06 ± 0.47^#^		0.026

The number of metastatic organs, number of metastatic lesions, number of previous systemic therapy and PRaG Cycles were described with Median (P25, P75) for abnormal distribution; other numerical variables were normally distributed and described with mean ± SD. The number of metastatic organs, number of metastatic lesions, number of previous systemic therapy and PRaG cycles, and ECOG score were compared with the Kruskal–Wallis Test among groups of CR+PR, SD, and PD; other normally distributed variables were compared with one-way ANOVA among groups of CR+PR, SD, and PD.

a Liver metastases were compared with the Chi-square test among groups of CR+PR, SD, and PD; other categorical variables were compared with the Fisher’s exact test among groups.

*Compared with the CR+PR group, the difference was statistically significant.

#Compared with the SD group, the difference was statistically significant.

**Figure 5:**
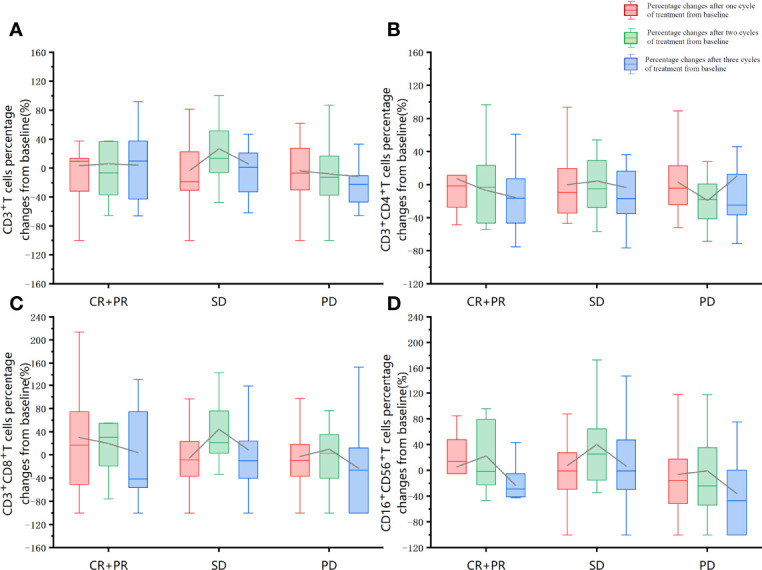
Lymphocyte subset percentage changes after treatment from baseline between the three groups (CR+PR, SD, PD). The red boxplot represents percentage changes after one cycle of treatment from baseline. The green boxplot represents percentage changes after two treatment cycles from baseline. The blue boxplot represents percentage changes after three cycles of treatment from baseline. The differences in the proportion of changes after the first cycle of treatment, after the second cycle of treatment, and after the third treatment cycle was compared separately between the three groups (CR+PR, SD, PD). The one-way ANOVA was used for the homogeneity of consistent variance, and the rank-sum test was used for the homogeneity of inconsistent variance. None of the other lymphocyte subset percentage changes showed statistical differences (*p* > 0.05). **(A)** CD3^+^T cells percentage changes from baseline. **(B)** CD3^+^CD4^+^T cells percentage changes from baseline. **(C)** CD3^+^CD8^+^T cells percentage changes from baseline. **(D)** CD16^+^CD56^+^T cells percentage changes from baseline.

## Discussion

Treatments for patients with metastatic solid cancers who failed previous standard systemic therapies are limited, especially for patients with a high tumor burden and multiple sites of metastases. Multisite radiotherapy in combination with pembrolizumab was studied in a phase I trial in heavily pretreated solid tumor patients, with ORR of 13.2%, median PFS of 3.1 months (95% CI, 2.9 to 3.4 months), and median OS of 9.6 months (95% CI, 6.5 months to undetermined) (5). In the present study, most of the patients had poor ECOG performance score of 2–3 and received a median of three previous lines of therapy. The ORR was 16.7% and DCR was 46.3% in the ITT population, and the median PFS of 4.0 months (95% CI, 3.3 to 4.8) and median OS of 10.5 months (95% CI, 8.7 to 12.2) in ITT population. TRAEs were reported in 70.0% of these subjects, while grade 3 or 4 events were reported in 12.0% of subjects. Notably, the NSCLC patient who have obtained CR in the present study failed previous single-agent PD-1 blockade immunotherapy, indicating the potential superior efficacy of the PRaG regimen. Altogether, all those findings strongly suggested that the PRaG regimen was safe and displayed potential clinical benefits in patients who failed the current standard treatments.

Radiotherapy may cause multiple pro-immunogenic changes within the TME, which convert cancer into an *in-situ* vaccine *via* releasing abundant levels of tumor-derived antigens, and GM-CSF was often used as a vaccine adjuvant (24, 25). GM-CSF can also enhance the antigen presentation by promoting the differentiation and activation of monocytes/M1 macrophages and DCs (26, 27). In addition, GM-CSF can upregulate HLA-DR expression and reverse the immune-suppressive effects of myeloid-derived suppressor cells (MDSCs) and regulatory T cells Tregs. In this regard, we hypothesized that the addition of GM-CSF may improve the therapeutic effects of radiotherapy in terms of enhanced tumor antigen presentation and recognition by tumor antigen specific T cells, which was likely further boosted by PD-1 immunotherapy, given that radiotherapy, GM-CSF, and PD-1 antibody treatment specifically targeted different components of the cancer-immunity cycle. But we should also pay attention to that in recent study tumor can induce CD45^+^ erythroid precursor cells (EPCs) subpopulation to differentiate into erythroid-differentiated myeloid cells (EDMCs). And tumor-derived GM-CSF directs EDMCs development from EPCs. EDMC can develop into MDSC-like subset, which damage the function of T cells and suppress immune activity (28). But the impact of exogenous GM-CSF on EDMCs amplification remains to be further investigated in cancer patients.

Moreover, multisite radiotherapy was suggested instead of single-site irradiation to expose sufficient tumor-associated antigens and decrease tumor burden (17, 23). But it was recognized that lymphocytes, in particular, the proliferating lymphocytes, were also radiosensitive, and their LD50 was 2 Gy and LD90 was 3 Gy (29). Of note, lymphocytopenia was an anti-PD-1/PD-L1 immunotherapy-related adverse event associated with efficacy (30). Large radiation fields, high RT doses, and multiple numbers of fractions were also risk factors for lymphocytopenia, primarily when RT was delivered by traditional fractionated external beam radiation therapy. In the present study, multiple irradiation cycles but with one distinct lesion irradiated each time and small volume each cycle might protect lymphocytes from damage. Tumor-draining lymph nodes (TDLNs) irradiation might lead to early short-term elimination of immune function due to lymphocyte depletion. Although it was not advisable for patients with node-negative disease, it should be beneficial for patients with extensive lymph nodes metastasis. In addition, the balance of immune cells in the invaded TDLN could shift to the regulatory pathway. It was necessary to eliminate Tregs, immunosuppressed DC and monocytes by irradiation, so that “healthy” blood derived immune cells could proliferate in the presence of systemic immune response.Immunostimulatory cytokines, such as GM-CSF, might help to restore such effector TDLN function (31).

The overall safety profile of PRaG therapy was acceptable and controllable. Continuous 14-day administration of GM-CSF was related to transient fever (38.0%) and G1–2 fatigue (16.0%). However, the incidence rates of all-grade TRAEs, or grade 3 and above events were comparable to those of other combination radiotherapy trials with PD-1/PD-L1 inhibitors (32). Early studies in lung cancers, including the KEYNOTE-001 and PACIFIC trials, demonstrated controllable toxicities with combined radiotherapy with PD-1 inhibitors (33, 34). However, with further analysis of KEYNOTE-001, patients who received radiotherapy had a higher incidence of pneumonia compared to those who did not (13% vs. 1%, *p* = 0.046). Still, no significant difference was shown in severe pulmonary toxicities. Similarly, the PACIFIC trial showed that the occurrence rate of severe TRAEs did not differ significantly between the durvalumab and the placebo arm (29.9% vs. 26.1%). At present, no evidence was shown that GM-CSF could increase the incidence of severe pneumonia in this study. Three patients experienced a transient decrease in pulse oxygen saturation after GM-CSF administration which was rarely reported in previous studies, and required further in-depth investigations.

Importantly, we intended to evaluate the potential prognostic factors at baseline in our study. Previous studies have shown that NLR can be a prognostic marker in certain cancer patients treated with PD-1 blockade (35–37). Yet, we did not find any association between NLR and PRaG therapeutic efficacy. In addition, baseline levels of lymphocyte subsets including CD4^+^T cells, CD8^+^T cells, NK cells, and B cells were not correlated with the response to PRaG treatment. In contrast, the CD4^+^/CD8^+^ ratio showed a significant correlation between the SD and PD groups. High CD3^+^T cells, CD3^+^CD4^+^T cells, CD3^+^CD8^+^T cells, and NK cell levels after one cycle of the PRaG regimen might be associated with better therapeutic effects (**Supplementary Figure 1**) but these still need to be confirmed with large sample size. It remains to be characterized in future studies regarding the dynamic changes of specific subsets such as CD4^+^Teff and Tregs.

Previous studies indicated that increased interleukin (IL)-6 was related to poor PFS in non-small cell lung carcinoma patients treated with anti-PD-1 inhibitors (38, 39). The activation of IL-6/STAT3 signaling pathway promotes tumorigenesis by increasing immunosuppressive myeloid-derived suppressor cells (MDSC) generation and inhibiting DC, NK, and T cell function in the TME (39). To this end, lower IL-6 levels or decreased IL-6 levels after two cycles of PRaG might be a potential predictor for improved therapeutic effect, but we did not observe significant correlation which might be due to the small sample size or detection sensitivity (**Supplementary Figure 3**). PD-L1 status, TMB, and MSI-H/dMMR were demonstrated to have certain predictive values of response to single-agent immunotherapy (2, 40). In particular, PD-L1 negative patients or those patients with MSS and low TMB might potentially benefit from the combined immune checkpoint inhibitors and radiotherapy, given the possible increased PD-L1 expression, elevated TMB upon radiation therapy. We have noticed that one patient obtained CR, and one got PR among thirteen NSCLC patients lacking driver gene mutations enrolled in this study. It is worth noting that the CR patient failed previous anti-PD-1 therapy (**Supplementary Figure 4**), and the PR patient had an immune non-responsive signature, including a lack of PD-L1 expression and low TMB. Moreover, among eight colorectal cancer patients, one patient was evaluated CR and one patient with MSS tumors had a PR (**Supplementary Figure 4**). Yet, further molecular profile analysis is needed to help identify potential predictive biomarkers for patients who benefit from this treatment modality. In the future, we will perform in-depth analysis of the mechanistic insights into the encouraging therapeutic efficacy of the PRaG regimen, in particular the patients that are largely insensitive to PD-1/PD-L1 inhibitor monotherapy and patients who failed prior immune checkpoint inhibitors therapy.

To our knowledge, this study was the first to investigate the efficacy and safety of multiple cycles of radiotherapy combined with PD-1 blockade and GM-CSF in patients with chemo-refractory metastatic cancers. The considerable therapeutic effects of the PRaG regimen were likely attributed to both local and systemic immunity triggered by irradiation, which was further boosted by PD-1 blockade and GM-CSF. The combinational effects might be related to the dose and fraction size of radiotherapy, combination timing schedule, the volumes and numbers of irradiated targets, the characteristics of primary and metastatic tumor types, and their intrinsic radio-sensitivity. Nevertheless, the current trial has several limitations. First, our study was carried out in a single study center with small sample size. A randomized, multi-center trial with a selected tumor type will be needed to validate the efficacy and safety of the PRaG regimen. Second, we could not characterize the precise action of mode of the combined therapy. Finally, it is of great interest to identify valuable biomarkers for probing the treatment efficacy and safety concerns in the era of precision medicine.

## Conclusion

In summary, the PRaG regimen was conducted in patients across multiple types of solid tumors with advanced stage of disease and metastases. Importantly, we did observe encouraging therapeutic effects of the PRaG treatment in certain fraction of patients who even failed previous single agent PD-1 blockade therapy. In this regard, the PRaG regimen was well tolerated with acceptable toxicity and may represent as a potential salvage treatment for patients with chemotherapy-refractory metastatic solid tumors.

## Data Availability Statement

The raw data supporting the conclusions of this article will be made available by the authors upon reasonable ask.

## Ethics Statement

The studies involving human participants were reviewed and approved by the Ethics Committee of The Second Affiliated Hospital of Soochow University. The patients/participants provided their written informed consent to participate in this study.

## Author Contributions

YK, XZ, MX, PX and LZh conceived and conducted the study, analyzed the results, and composed the manuscript. YK, XZ, MX have contributed equally to this work and share first authorship. YM LZo, QP, JZ, WZ, YP, JY, CZ and YL helped collect the data. QG and GC performed an imaging evaluation. ZX, HW and CS participated in manuscript review and helped with the statistical analyses. All authors contributed to the article and approved the submitted version.

## Funding

This study was supported by the National Natural Science Foundation of China(82171828), the Key R&D plan of Jiangsu Province (Social Development, BE2021652), Suzhou Radiotherapy Clinical Medical Center (Szlcyxzx202103), Open project of the State Key Laboratory of Radiology and Radiation Protection of Soochow University (GZK1202014), Open Project of Provincial Key Laboratory of Soochow University (KJS1961), the Subject construction support project of the Second Affiliated Hospital of Soochow University (XKTJ-RC202001, XKTJ-HRC20210011), the Suzhou Science and Technology Development Plan (SYS2020143), Chinese Society of Clinical Oncology Research Foundation of Beijing (Y-XD202002/ZB-0015), Wu Jieping Medical Foundation (320.6750.2021-01-12), Open project of Provincial Key Laboratory of Soochow University (KJS1961), and the special project of “Technological Innovation” project of CNNC Medical Industry Co. Ltd (ZHYLTD2021001), Suzhou Science and Education Health Project (KJXW2021018) Postgraduate Research & Practice Innovation Program of Jiangsu Province (SJCX22_1508).

## Conflict of Interest

Author ZX was employed by ICON Plc.

The remaining authors declare that the research was conducted in the absence of any commercial or financial relationships that could be construed as a potential conflict of interest.

## Publisher’s Note

All claims expressed in this article are solely those of the authors and do not necessarily represent those of their affiliated organizations, or those of the publisher, the editors and the reviewers. Any product that may be evaluated in this article, or claim that may be made by its manufacturer, is not guaranteed or endorsed by the publisher.
