# Supplementary material for: PD-1 Inhibitor Combined With Radiotherapy and GM-CSF (PRaG) in Patients With Metastatic Solid Tumors: An Open-Label Phase II Study
Source: Front Immunol. 2022 Jul 8;13:952066. doi: 10.3389/fimmu.2022.952066 (PMC9304897; doi:10.3389/fimmu.2022.952066)
Supplement: Supplementary file 1 [file DataSheet_1.pdf]

Supplementary Material

Supplement 1： Details of study design

OBJECTIVE

Primary Objective

To observe the Objective Response Rate (ORR) of radiotherapy combined with PD-1 inhibitor and GM-CSF in intent-to-treat (ITT) metastatic solid tumor patients.

Secondary Objectives

- Disease Control Rate (DCR) was defined as the percentage of patients having a complete response(CR), partial response(PR), or stable disease(SD) ⩾12 weeks from the start of enrollment.
- Progression-Free Survival (PFS) was calculated from the enrollment date to the first progressive disease, death, or censored at the last clinical follow-up.
- Overall Survival (OS) was calculated from the enrollment date to date last known alive or death.
- Toxicity

METHODOLOGY

Entry Criteria

To be eligible for participation in this trial, the subject must:

1. Aged ⩾18 years old;

2. diagnosed with advanced solid cancer with multiple metastases, progressed after at least first-line chemotherapy recommended by the NCCN guidelines, and have at least three measurable tumor lesions (>1 cm);

3. Acceptable hematopoietic function and general condition (white blood cells >2.5×10^9/L, platelets >50×10^9/L, liver function glutamic-pyruvic transaminase and glutamic-oxaloacetic transaminase not exceeding 2.5 times the normal upper limit);

4. No congestive heart failure, unstable angina pectoris, and unstable arrhythmia occurred in the past six months;

5. Eastern Cooperative Oncology Group (ECOG) score of 0-3 and life expectancy of more than three months;

6. No history of immune diseases;

7. Abide by the plan during the study period;

8. Sign written consent.

Subject Exclusion Criteria

1. Pregnant or lactating women;

2. Patients diagnosed with other malignant diseases in the past five years, except for cured skin cancer and cervical carcinoma in situ;

3. The clinical severity of uncontrolled epilepsy, central nervous system disease, or mental disorder may hinder the signing of informed consent or affect the patient's compliance with medication.

4. Severe (e.g., active) heart disease, such as symptomatic coronary heart disease, New York Heart Association (NYHA) class II or more severe congestive heart failure or severe arrhythmia requiring drug intervention, or a history of myocardial infarction in the last 12 months;

5. Organ transplantation requires immunosuppressive therapy;

6. Known major active infections, or significant blood, kidney, metabolism, gastrointestinal, endocrine or metabolic disorders, or other uncontrolled severe concomitant diseases judged by the researchers;

7. The baseline blood routine does not meet the following criteria: hemoglobin (⩾ 70g/L); absolute neutrophil count (ANC) (⩾ 1.0×10^9/L); platelet (>50×10^9/L); ALT, AST (⩽ 2.5 times normal upper limit value);

8. Anaphylaxis to any research drug ingredients.

9. Patients with a history of immunodeficiency, including HIV positive or with other acquired or congenital immunodeficiency disorders, or with a history of organ transplantation, or with other immune-related diseases requiring long-term oral hormone therapy;

10. In the period of acute and chronic tuberculosis infection (T-spot test positive, chest X-ray suspicious tuberculosis focus patients);

11. Other situations which are not suitable for enrollment.

Sample size

The sample size of 50 subjects is not designed to make explicit power and Type I error considerations for a hypothesis test. It is primarily chosen to obtain a preliminary assessment of antitumor activity with a certain degree of precision.

A sample size of 42 from a population of 50 produces a two-sided 95% confidence interval with a precision (half-width) of 0.0500 when the ORR is near 0.2000. Considering the drop-out rate of 10%, additional 5 subjects are needed and total number of subjects is at least 47.

Trial Treatments Radiotherapy

Patients will be mainly positioned supine, but prone positioning is allowed if needed. Multiple organ sites may be involved, and it is up to the treating physician to decide what tumor localization to treat. SBRT or HFRT is performed for a suitable metastatic lesion in each triple treatment cycle.

The use of an immobilization device is obligated to ensure set-up reproducibility. Immobilization may be performed under each department's routine practice for subject positioning. A 4D-CT scan in treatment position with 5 mm slice thickness will be made in lung, liver, and pancreatic tumors.

The Gross Tumor Volume (GTV) will encompass the designated tumor using the appropriate window-level setting depending on the treated site, e.g., lung window level in case of a lung tumor. The Clinical Tumor Volume (CTV) will encompass regions at risk for the microscopic extension. Participating institutes are free to define their GTV to CTV margins. The Planning Tumor Volume (PTV) will be generated by expanding either the CTV or GTV using individualized margins in case of tumor motion.

Organs at risk

The involved organs at risk located near the designated tumor will be delineated. The dose limits of organs at risk refer to the following table.

Three Fractions				Timmerman
**Serial Tissue**	**Volume**	**Volume Max (Gy)**	**Max Point Dose (Gy)****	**Endpoint (≥Grade 3)**
Optic Pathway	<0.2 cc	15.3 Gy (5.1 Gy/fx)	17.4 Gy (5.8 Gy/fx)	neuritis
Cochlea			17.1 Gy (5.7 Gy/fx)	hearing loss
Brainstem (not medulla)	<0.5 cc	18 Gy (6 Gy/fx)	23.1 Gy (7.7 Gy/fx)	cranial neuropathy
Spinal Cord and medulla	<0.35 cc<br><1.2 cc	18 Gy (6 Gy/fx)<br>12.3 Gy (4.1 Gy/fx)	21.9 Gy (7.3 Gy/fx)	myelitis
Spinal Cord Subvolume (5-6 mm above and below level treated per Ryu)	<10% of subvolume	18 Gy (6 Gy/fx)	21.9 Gy (7.3 Gy/fx)	myelitis
Cauda Equina	<5 cc	21.9 Gy (7.3 Gy/fx)	24 Gy (8 Gy/fx)	neuritis
Sacral Plexus	<5 cc	22.5 Gy (7.5 Gy/fx)	24 Gy (8 Gy/fx)	neuropathy
Esophagus*	<5 cc	17.7 Gy (5.9 Gy/fx)	25.2 Gy (8.4 Gy/fx)	stenosis/fistula
Brachial Plexus	<3 cc	20.4 Gy (6.8 Gy/fx)	24 Gy (8 Gy/fx)	neuropathy
Heart/Pericardium	<15 cc	24 Gy (8 Gy/fx)	30 Gy (10 Gy/fx)	pericarditis
Great vessels	<10 cc	39 Gy (13 Gy/fx)	45 Gy (15 Gy/fx)	aneurysm
Trachea and Large Bronchus*	<4 cc	15 Gy (5 Gy/fx)	30 Gy (10 Gy/fx)	stenosis/fistula
Bronchus- smaller airways	<0.5 cc	18.9 Gy (6.3 Gy/fx)	23.1 Gy (7.7 Gy/fx)	stenosis with atelectasis
Rib	<1 cc	28.8 Gy (9.6 Gy/fx)	36.9 Gy (12.3 Gy/fx)	Pain or fracture
Skin	<10 cc	30 Gy (10 Gy/fx)	33 Gy (11 Gy/fx)	ulceration
Stomach	<10 cc	16.5 Gy (5.5 Gy/fx)	22.2 Gy (7.4 Gy/fx)	ulceration/fistula
Bile duct			35.7 Gy (11.9 Gy/fx)	stenosis
Duodenum*	<5 cc<br><10 cc	16.5 Gy (5.5 Gy/fx)<br>11.4 Gy (3.8 Gy/fx)	22.2 Gy (7.4 Gy/fx)	ulceration
Jejunum/Ileum*	<5 cc	17.7 Gy (5.9 Gy/fx)	25.2 Gy (8.4 Gy/fx)	enteritis/obstruction
Colon*	<20 cc	24 Gy (8 Gy/fx)	28.2 Gy (9.4 Gy/fx)	colitis/fistula
Rectum*	<20 cc	24 Gy (8 Gy/fx)	28.2 Gy (9.4 Gy/fx)	proctitis/fistula
Ureter			48.9 (16.3 Gy/fx)	stenosis
Bladder wall	<15 cc	16.8 Gy (5.6 Gy/fx)	28.2 Gy (9.4 Gy/fx)	cystitis/fistula
Penile bulb	<3 cc	21.9 Gy (7.3 Gy/fx)	42 Gy (14 Gy/fx)	impotence
Femoral Heads (Right & Left)	<10 cc	21.9 Gy (7.3 Gy/fx)		necrosis
Renal hilum/vascular trunk	<2/3 volume	18.6 Gy (6.2 Gy/fx)		malignant hypertension
**Parallel Tissue**	**Critical Volume (cc)**	**Critical Volume Dose Max (Gy)**		**Endpoint (≥Grade 3)**
Lung (Right & Left)	1500 cc	10.5 Gy (3.5 Gy/fx)		Basic Lung Function
Lung (Right & Left)	1000 cc	11.4 Gy (3.8 Gy/fx)		Pneumonitis
Liver	700 cc	17.1 Gy (5.7 Gy/fx)		Basic Liver Function
Renal cortex (Right & Left)	200 cc	14.4 Gy (4.8 Gy/fx)		Basic renal function

Dose specification and treatment planning

Patients will be treated with hypofractionated radiotherapy/stereotactic body radiation of metastatic lesions at doses of 5Gy or 8Gy for three fractions.

Dose specification will be performed according to local policies, and dose inhomogeneity in the PTV is allowed. Volumetric Modulated Arc Therapy (VMAT) or static Intensity Modulated Radiotherapy (IMRT) will be used. Ninety-five percent of the PTV should receive 100% of the prescribed dose.

PD-1 inhibitor

PD-1 inhibitor intravenously administered once within one week after completion of radiotherapy.

Drug	Dose	Dose Frequency	Route of Administration	Treatment Period
Pembrolizumab (Merck, USA)	200mg	Q3W	IV infusion	3weeks
Sintilimab (Innovent Biologics, China)	200mg	Q3W	IV infusion	3weeks
Camrelizumab (Hengrui Pharmaceuticals, China)	200mg	Q3W	IV infusion	3weeks
Toripalimab (Junshi Biosciences, China)	240mg	Q3W	IV infusion	3weeks
Tislelizumab (Beigene, China)	200mg	Q3W	IV infusion	3weeks

GM-CSF

GM-CSF (Molgramostim, Topleucon, Xiamen Amoytop Biotech, China) 200 µg injection subcutaneously daily for two weeks started within 24 hours after the last fraction of radiotherapy.

Maintenance treatment

The PRaG course will repeat every 21 days for at least two cycles until no appropriate lesions for irradiation, reach the tolerance dose of normal tissues, or have severe side effects of radiotherapy. After triple-combination therapy discontinuation, patients without disease

progression will continue PD-1 inhibitor monotherapy until disease confirmed progression or unacceptable toxicity. It is allowed to continue the treatment after the first evaluation of progression, but it needs to be re-evaluated after one month. If progression is confirmed after secondary evaluation, the patient will not continue the treatment.

Assessing

RECIST version 1.1 will be used to assess tumor response. The irradiated lesions should not be included in the target lesions at the evaluation time. An independent radiologist will evaluate the imaging data. The evaluation will take place every eight weeks.

RECIST1.1 was published in the European Journal of Cancer:

Eisenhauer EA, Therasse P, Bogaerts J, Schwartz LH, Sargent D, Ford R, Dancey J, Arbuck S, Gwyther S, Mooney M, Rubinstein L, Shankar L, Dodd L, Kaplan R, Lacombe D, Verweij J. New response evaluation criteria in solid tumors: revised RECIST guideline (version 1.1). Eur J Cancer. 2009 Jan;45(2):228-47.

iRECIST was published in The Lancet Oncology:

Seymour L, Bogaerts J, Perrone A, Ford R, Schwartz LH, Mandrekar S, Lin NU, Litière S, Dancey J, Chen A, Hodi FS, Therasse P, Hoekstra OS, Shankar LK, Wolchok JD, Ballinger M, Caramella C, de Vries EGE; RECIST working group. iRECIST: guidelines for response criteria for use in trials testing immunotherapeutics. Lancet Oncol. 2017 Mar;18(3): e143-e152.

Safety

Adverse events will be collected from patients signed informed consent forms (ICF) until 90 days post last study treatment, using Common Terminology Criteria for Adverse Events (CTCAE), version 4.0, with investigator assessment to causality.

Common Terminology Criteria for Adverse Events V4.0 (CTCAE)

The descriptions and grading scales found in the revised NCI Common Terminology Criteria for Adverse Events (CTCAE) version 4.0 will be utilized for adverse event reporting. Any adverse event that changes CTCAE grade over a given episode will have each change of grade recorded on the adverse event case report forms/worksheets.

Patients will be examined regularly during and after treatment to evaluate the curative effect and prognosis. The examination items and time points are shown in the table below.

	Baseline	After one course of treatment	PRaG treatment period (every eight weeks)	Maintenance treatment period (every two months)
Physical examination	X	X	X	X
Blood routine examination	X	X	X	X
Hepatorenal function	X	X	X	X
Tumor marker	X	X	X	X
Thyroid function	X	X	X	X
Myocardial enzyme	X	X	X	X
Glycated hemoglobin	X	X	X	X
ECG	X	X	X	X
Chest computed tomography(CT)	X		X	X
Abdominal CT	X		X	X
Pelvic CT	X		X	CT/MRI
Cranial MR (patients with brain metastasis)	X		X	X
Quality of life evaluation	X	X	X	X

Supplementary Figures and Tables

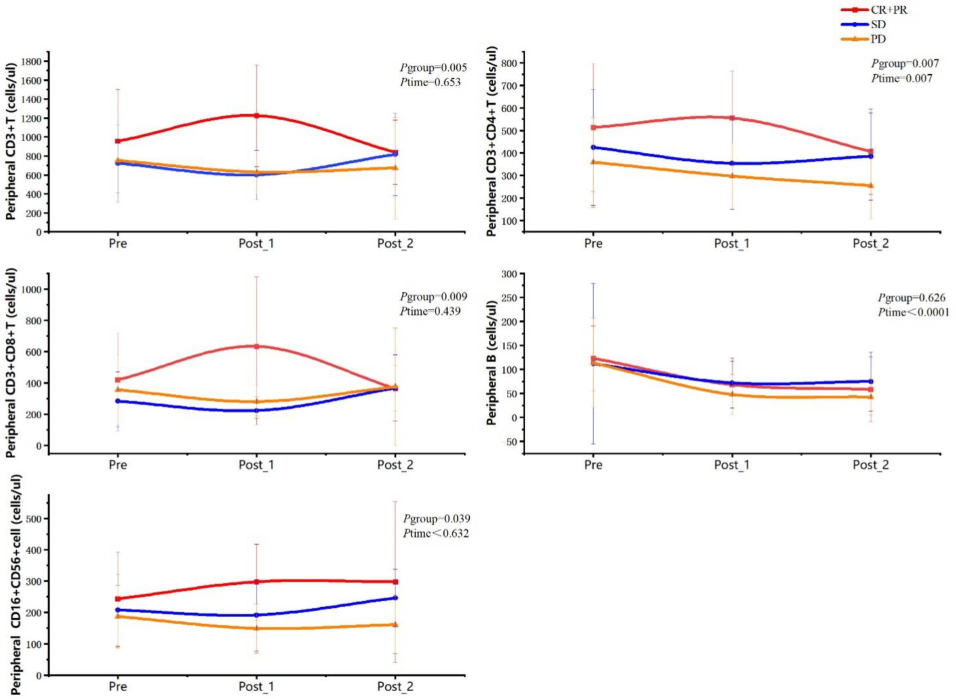

Supplementary Figure 1

Mean lymphocyte subset number changes of Pre(baseline), Post_1(after one cycle of PRaG treatment), and Post_2(after two cycles of PRaG treatment) of the three groups (CR+PR, SD, PD). The Redline represents the CR+PR group, blue represents the SD group, and orange represents the PD group. The repeated measures analysis of variance (RM ANOVA) was used for the homogeneity of consistent variance, and the rank-sum test was used for the homogeneity of inconsistent variance. Pre, Post_1, and Post_2 numbers were compared separately ($p_{time} <$ 0.05 means if any of the three groups has different changes by Pre, Post_1, and Post_2 numbers). The CR+PR, SD, and PD groups were compared separately by each treatment time ($p_{group} < 0.05$ means if any one of the three treatment time has differently changed by the CR+PR, SD, and PD group).

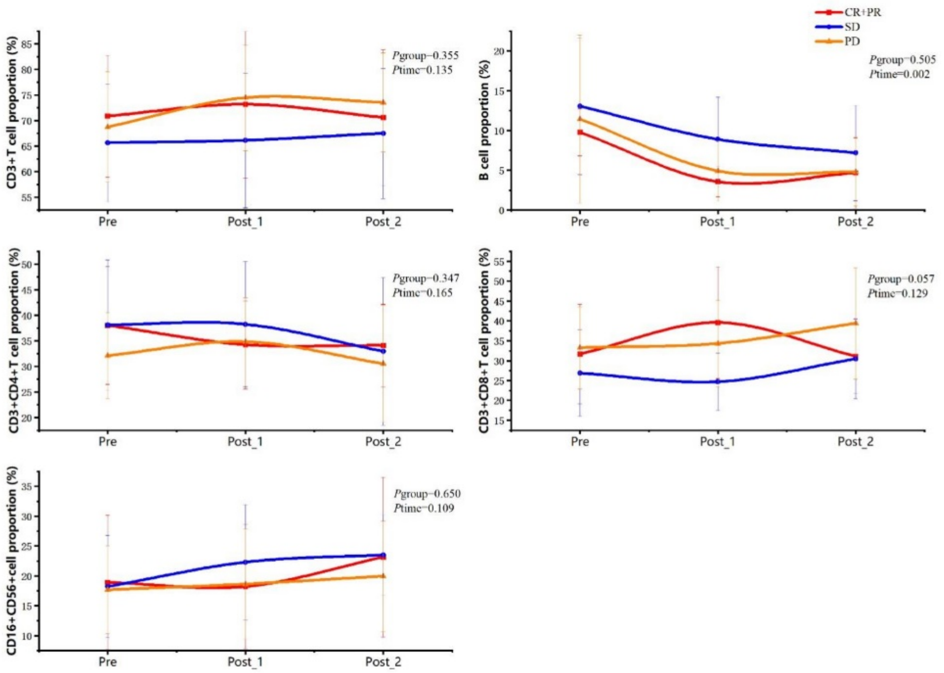

Supplementary Figure 2

Mean lymphocyte subset proportion changes of Pre(baseline), Post_1(after one cycle of PRaG treatment), and Post_2(after two cycles PRaG treatment) of the three groups (CR+PR, SD, PD). The Redline represents the CR+PR group, blue represents the SD group, and orange represents the PD group. The RM ANOVA was used for the homogeneity of consistent variance, and the rank-sum test was used for the homogeneity of inconsistent variance. Pre, Post_1, and Post_2 numbers were compared separately ($p_{time} < 0.05$ means if any of the three groups has differently changes by Pre, Post_1, and Post_2 numbers). CR+PR, SD, and PD groups were compared separately by each treatment time ($p_{group} < 0.05$ means if any one of the three treatment timed has differently changed by the CR+PR, SD, and PD group).

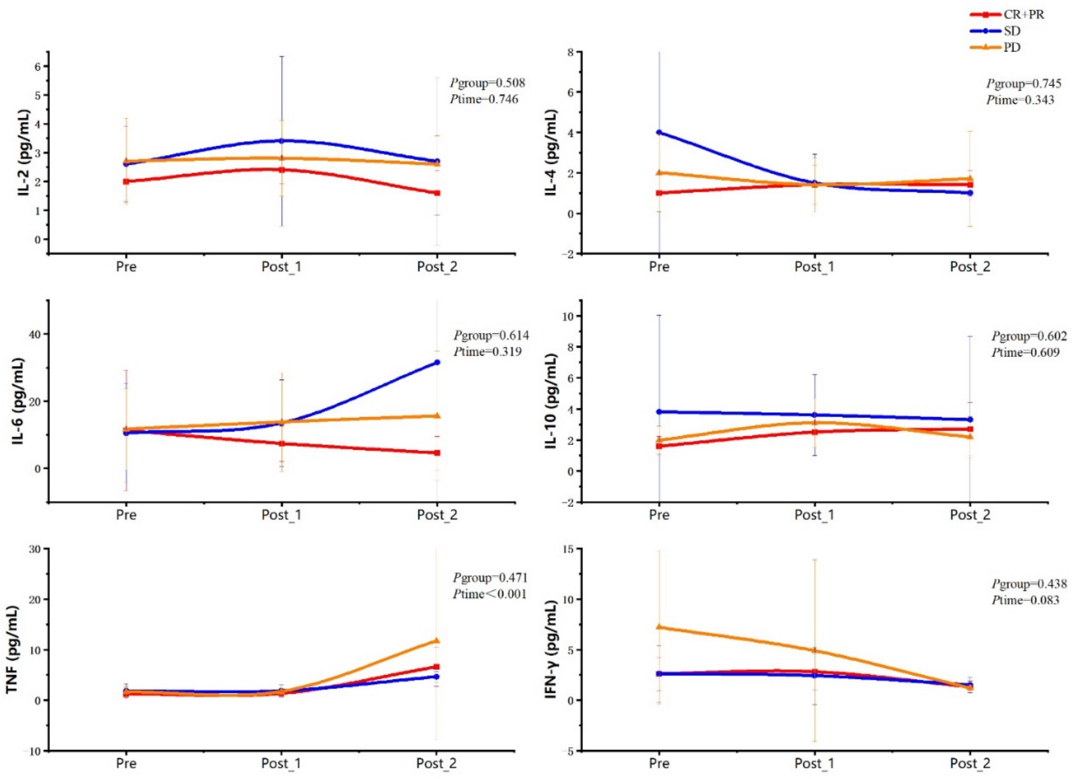

Supplementary Figure 3

Mean serum cytokine changes of Pre(baseline), Post_1(after one cycle of PRaG treatment), and Post_2(after two cycles of PRaG treatment) of the three groups (CR+PR, SD, PD). The Redline represents the CR+PR group, blue represents the SD group, and orange represents the PD group. The RM ANOVA was used for the homogeneity of consistent variance, and the rank-sum test was used for the homogeneity of inconsistent variance. Pre, Post_1, and Post_2 numbers were compared separately ($p_{time}<0.05$ means if any of the three groups has different changes by Pre, Post_1, and Post_2 numbers). CR+PR, SD, and PD groups were compared separately by each treatment time ($p_{group}<0.05$ means if any one of the three treatment times has differently changed by the CR+PR, SD, and PD group).

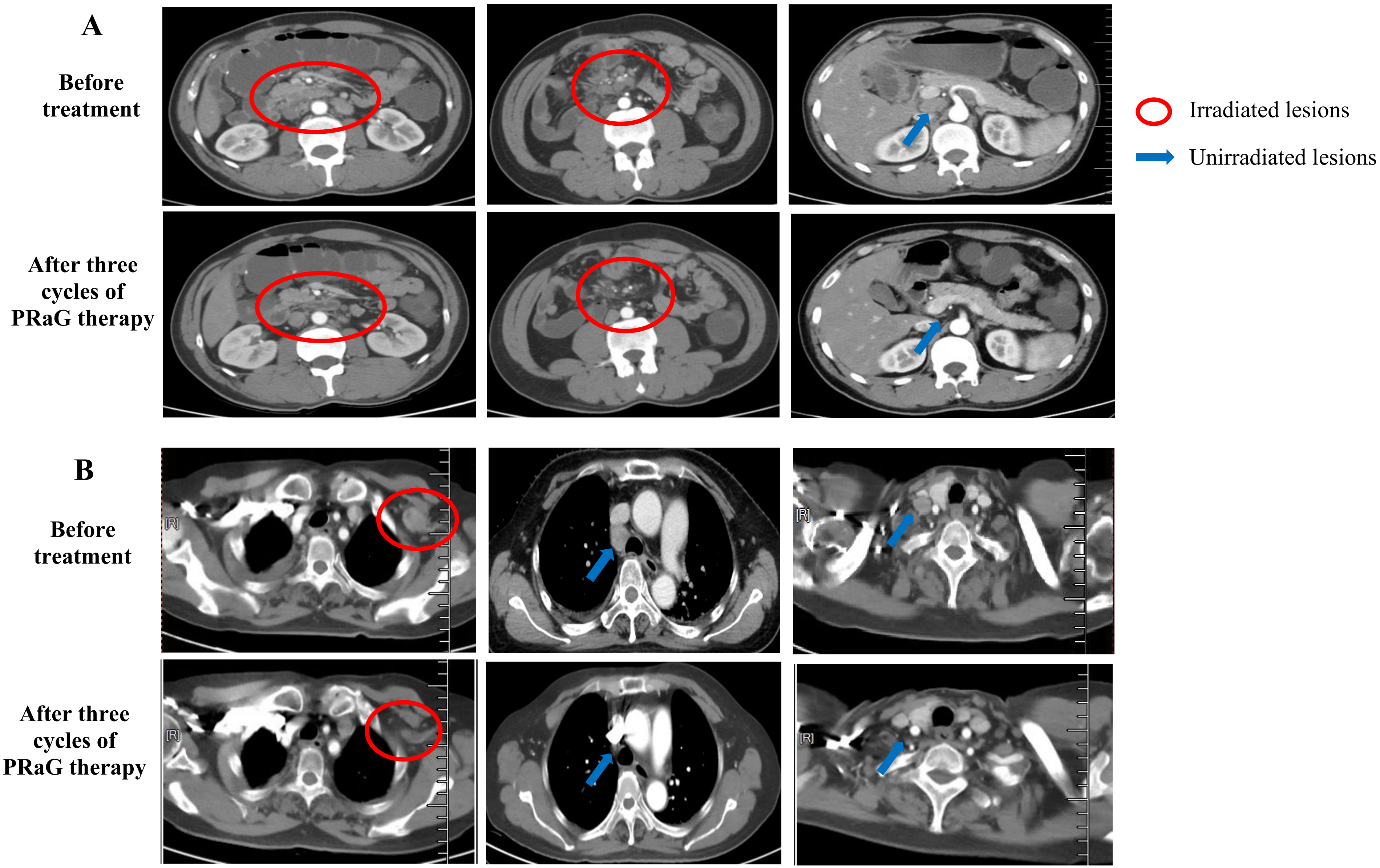
A
Before treatment
After three cycles of PRaG therapy
Irradiated lesions
Unirradiated lesions
B
Before treatment
After three cycles of PRaG therapy
[R]

Supplementary Figure 4

CT scans of two patients before and after three cycles of PRaG therapy. Red circles represented the irradiated lesions, and blue arrows represented the unirradiated lesions. (A)The CT images of a metastatic MSS colon cancer patient showed that the irradiated and unirradiated lesions shrunk or even disappeared. (B)An NSCLC patient who failed previous anti-PD-1 therapy was successfully rechallenged by PRaG therapy. This patient's irradiated and unirradiated lymph nodes were significantly smaller or even disappeared than before treatment.

Supplementary Table 1 Interest TRAEs related to each of the three treatments

	Radiotherapy				PD-1 inhibitor				GM-CSF			
TRAEs	Gr1	Gr2	Gr3	Gr4	Gr1	Gr2	Gr3	Gr4	Gr1	Gr2	Gr3	Gr4
Fever	0	0	0	0	0	0	0	0	11(22.0%)	7(14.0%)	1(2.0%)	0
Thyroid dysfunction	0	0	0	0	3(6.0%)	12(24.0%)	0	0	0	0	0	0
Liver dysfunction	0	0	0	0	4(8.0%)	1(2.0%)	1(2.0%)	0	0	0	0	0
Rash	0	0	0	0	2(4.0%)	0	0	0	5(10.0%)	0	0	0
Vomiting	3(6.0%)	1(2.0%)	0	0	0	0	0	0	3(6.0%)	0	0	0
Pneumonia/Pneumonitis	0	5(10.0%)	0	1(2.0%)	0	0	1(2.0%)	0	0	0	0	0
Myocarditis	0	0	0	0	0	1(2.0%)	0	0	0	0	0	0
Uveitis	0	0	0	0	0	1(2.0%)	0	0	0	0	0	0
Pruritus	0	0	0	0	2(4.0%)	0	0	0	0	0	0	0
Decrease in pulse oxygen	0	0	0	0	0	0	0	0	2(4.0%)	1(2.0%)	0	0
Leukocytosis	0	0	0	0	0	0	0	0	5(10.0%)	0	0	0

Supplementary Table 2 Treatment response was classified by primary tumor sites

**Parameter**	**Lung (n=13)**	**Colorectum (n=8)**	**Breast (n=5)**	**Gastro (n=5)**	**Cervix (n=4)**	**Esophagus (n=4)**	**Ovary (n=4)**	**Head and Neck (n=4)**	**Liver (n=2)**	**Soft tissue (n=2)**	**Kidney (n=1)**	**Vulva (n=1)**	**Bile duct (n=1)**
Best overall responses													
Complete response	1(7.7)	1(12.5)	0(0.0)	1(20.0)	0(0.0)	0(0.0)	0(0.0)	0(0.0)	0(0.0)	0(0.0)	0(0.0)	0(0.0)	0(0.0)
Partial response	1(7.7)	1(12.5)	1(20.0)	1(20.0)	0(0.0)	1(25.0)	0(0.0)	0(0.0)	0(0.0)	1(50.0)	0(0.0)	0(0.0)	0(0.0)
Stable disease	5(38.5)	3(37.5)	1(20.0)	0(0.0)	1(25.0)	1(25.0)	1(25.0)	2(50.0)	0(0.0)	0(0.0)	1(100.0)	1(100.0)	0(0.0)
Progressive disease	5(38.5)	3(37.5)	2(40.0)	2(40.0)	2(50.0)	2(50.0)	2(50.0)	2(50.0)	2(100.0)	0(0.0)	0(0.0)	0(0.0)	1(100.0)
Not evaluable	1(7.7)	0(0.0)	1(20.0)	1(20.0)	1(25.0)	0(0.0)	1(25.0)	0(0.0)	0(0.0)	1(50.0)	0(0.0)	0(0.0)	0(0.0)
ORR	2(15.4)	2(25.0)	1(20.0)	2(40.0)	0(0.0)	1(25.0)	0(0.0)	0(0.0)	0(0.0)	1(50.0)	0(0.0)	0(0.0)	0(0.0)
DCR	7(53.8)	5(62.5)	2(40.0)	2(40.0)	1(25.0)	2(50.0)	1(25.0)	2(50.0)	0(0.0)	1(50.0)	1(100.0)	1(100.0)	1(100.0)
